# Laser‐Induced Graphene‐Based Sensors in Health Monitoring: Progress, Sensing Mechanisms, and Applications

**DOI:** 10.1002/smtd.202400118

**Published:** 2024-04-10

**Authors:** Zihao Li, Libei Huang, Le Cheng, Weihua Guo, Ruquan Ye

**Affiliations:** ^1^ Department of Chemistry State Key Laboratory of Marine Pollution City University of Hong Kong Kowloon Hong Kong 999077 China; ^2^ Division of Science, Engineering and Health Study School of Professional Education and Executive Development The Hong Kong Polytechnic University (PolyU SPEED) Kowloon Hong Kong 999077 China; ^3^ City University of Hong Kong Shenzhen Research Institute Shenzhen 518057 China

**Keywords:** health monitoring, laser‐induced graphene, sensing mechanisms, wearable sensing devices

## Abstract

The rising global population and improved living standards have led to an alarming increase in non‐communicable diseases, notably cardiovascular and chronic respiratory diseases, posing a severe threat to human health. Wearable sensing devices, utilizing micro‐sensing technology for real‐time monitoring, have emerged as promising tools for disease prevention. Among various sensing platforms, graphene‐based sensors have shown exceptional performance in the field of micro‐sensing. Laser‐induced graphene (LIG) technology, a cost‐effective and facile method for graphene preparation, has gained particular attention. By converting polymer films directly into patterned graphene materials at ambient temperature and pressure, LIG offers a convenient and environmentally friendly alternative to traditional methods, opening up innovative possibilities for electronic device fabrication. Integrating LIG‐based sensors into health monitoring systems holds the potential to revolutionize health management. To commemorate the tenth anniversary of the discovery of LIG, this work provides a comprehensive overview of LIG's evolution and the progress of LIG‐based sensors. Delving into the diverse sensing mechanisms of LIG‐based sensors, recent research advances in the domain of health monitoring are explored. Furthermore, the opportunities and challenges associated with LIG‐based sensors in health monitoring are briefly discussed.

## Graphical abstract

This work summarizes the development of laser‐induced graphene sensors for health monitoring, which includes synthetic strategies for sensor fabrications, sensing mechanisms for different signals, and applications in various fields pertaining to health. Opportunities and challenges associated with laser‐induced graphene sensors for health monitoring are also discussed.

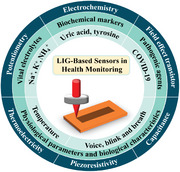

## 1 Introduction

The number of deaths from non‐communicable diseases, such as cardiovascular and chronic respiratory diseases, has risen sharply with population growth and rising standards of living.^[^ ^1^ ^]^ The prevalence of numerous diseases poses a severe threat to the health and even the lives of people around the world. As the leading cause of death, cardiovascular diseases killed ≈17.9 million people in 2019, according to WHO's World Health Statistics 2023 reports.^[^ ^2^ ^]^ Nevertheless, with advances in wearable sensing device technology, many diseases can be prevented by monitoring with low‐cost wearable microsensors,^[^ ^3^ ^]^ such as airflow sensors for respiratory disease monitoring,^[^ ^4^, ^5^ ^]^ heart rate and blood oxygen saturation sensors for detection of cardiovascular diseases.^[^ ^6^ ^]^

In the realm of micro‐sensing, graphene‐based sensors stand out as promising contenders.^[^ ^7^ ^]^ Graphene's unique properties, such as high specific surface area,^[^ ^8^ ^]^ excellent electrical conductivity,^[^ ^9^ ^]^ biocompatibility^[^ ^10^ ^]^ and chemical stability,^[^ ^11^ ^]^ position it as a highly anticipated material for health surveillance sensors. Laser‐induced graphene (LIG) technology is a cutting‐edge process for graphene preparation and has become one of the mainstream preparation processes for graphene‐based sensors.^[^ ^12^ ^]^ This technology can be carried out at ambient temperature and pressure, using a common laser to directly convert various precursors into graphene materials.^[^ ^13^ ^]^ Compared with traditional vapor deposition and wet chemical methods,^[^ ^14^, ^15^ ^]^ this technology avoids the reaction conditions of high temperature and pressure control, high purity gases, and produces a minimal amount of waste, thus having unrivaled advantages over a wide range of preparation methods.^[^ ^16^ ^]^ In addition, LIG technology combines graphene synthesis and pattern control as a whole, and can directly print electronic devices with desired patterns, further simplifying the productization route. Therefore, the integration of LIG‐based sensors into health monitoring systems holds significant potential for revolutionizing the way we approach preventive healthcare.

Recent advancements in LIG‐based materials have significantly broadened their applications in health monitoring.^[^ ^17^, ^18^ ^]^ Noteworthy are the developments in multi‐functional LIG‐based sensors that now enable the precise monitoring of a range of physiological signals and biomarkers,^[^ ^19^, ^20^ ^]^ crucial for early disease detection and management. Studies exploring the integration of LIG into wearable technology have demonstrated its potential for real‐time health assessment,^[^ ^21^, ^22^, ^23^ ^]^ thereby enhancing patient care through non‐invasive methods. Such progress reflects the growing capabilities of LIG‐based sensors in addressing the complex needs of modern healthcare diagnostics.

**Figure 1:**
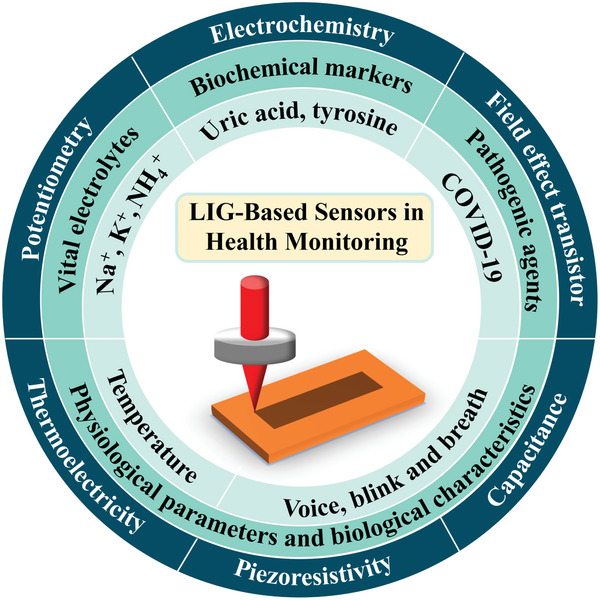
LIG‐based multiple sensing mechanisms for health monitoring.

To commemorate the ten‐year milestone since the discovery of LIG in 2014,^[^ ^24^ ^]^ this review commences by providing a brief overview of the development of LIG and LIG‐based sensors over this decade. Then, we will comprehensively delve into the various sensing mechanisms employed by LIG‐based sensors and elucidate their recent breakthroughs in the realm of health monitoring, the main content of which is shown in **Figure** **1**. Finally, a concise analysis of research gaps and outlook sheds light on the promising directions for the ongoing evolution of LIG‐based health monitoring sensors.

## 2 Development of LIG Technology and LIG‐based Sensors

Over the past decade, LIG has rapidly evolved from a new laboratory discovery to a cutting‐edge field of interdisciplinary research. This section focuses on a brief review of the development of LIG technology and LIG‐based sensors during this decade. In 2014, the discovery of LIG marked a breakthrough in materials science. For the first time, researchers achieved efficient preparation of graphene by direct laser irradiation of polyimide (PI) films.^[^ ^24^ ^]^ This method is simple, fast, and inexpensive, without the need for complex chemical etching or high‐temperature heat treatment, offering the possibility of large‐scale production and broad‐field applications.

In the following decade, the research and application of LIG experienced rapid development and expansion. Early on, LIG was mainly studied for use in electronic and energy storage devices,^[^ ^25^, ^26^, ^27^ ^]^ such as supercapacitors and batteries, because of its excellent electrical conductivity and mechanical strength. Chen et al. demonstrated an in‐situ method for creating superhydrophobic fluorine‐doped graphene, enabling the fabrication of a droplet‐based electricity generator with exceptional power conversion efficiency and operational stability.^[^ ^28^ ^]^ Another research introduced a novel approach for fabricating fluorine‐doped LIG, highlighting its application in developing Joule deicing heaters with enhanced performance.^[^ ^29^ ^]^ This method offers an easy pathway for preparing flexible devices with high performance based on LIG. These developments demonstrate the versatility of LIG in creating practical and efficient solutions for energy generation and the preparation of high‐performance electronic devices. As research progressed, scientists began to look for ways to improve the properties of LIG, including improving electrical conductivity, increasing surface area, and enhancing the mechanical stability of the material.^[^ ^30^ ^]^ The multi‐functionalization of LIG has been realized, including its use in various applications such as bactericidal,^[^ ^31^ ^]^ anti‐viral,^[^ ^32^ ^]^ nitrate reduction,^[^ ^33^, ^34^ ^]^ saltwater disinfection,^[^ ^35^ ^]^ sensing.^[^ ^36^ ^]^ As for LIG‐based sensing, Hou et al. presented a humidity sensor coated with LIG on a hollow‐core optical fiber with a sensitivity of 0.187 dB/% RH over a wide range (5‐95% RH), demonstrating the material's adaptability in humidity sensing.^[^ ^37^ ^]^ Another study proposed a flexible pressure sensor with high spatial resolution and minimal crosstalk interference by a two‐step laser processing that optimizes pixel interconnects.^[^ ^38^ ^]^ This advancement significantly enhances pressure sensing on soft surfaces, demonstrating LIG's potential in high‐resolution tactile pattern recognition and medical applications. These examples underscore LIG's expanding role in the sensing domain, setting the stage for its integration into health monitoring systems.

In recent years, the use of LIGs in health monitoring has been of particular interest.^[^ ^39^, ^40^ ^]^ LIG‐based sensors have been used to monitor a variety of physiological signals in the human body, such as heart rate, respiration, and even biomarkers in sweat. Its applications in biosensors, environmental monitoring, and food safety demonstrate its great potential for promoting innovation in healthcare. In addition, its use in smart wearable devices is growing, laying the groundwork for its use in real‐time human health monitoring.^[^ ^41^, ^42^ ^]^ Central to unlocking these diverse health monitoring applications is the process of laser‐induced graphene synthesis, which intricately depends on substrate choice, laser light characteristics, and the incorporation of additives. The preparation of LIG begins with selecting a carbon‐rich substrate, which is then subjected to controlled laser irradiation to induce local graphene transformation.

Firstly, the choice of substrate material influences the electrical properties, thermal stability, and biocompatibility of LIG. Initially, research focused on PI films, and researchers gradually explored a wider variety of substrate materials, such as paper and wood,^[^ ^43^ ^]^ to meet the needs of different applications. The original LIG substrate material, PI, makes LIG‐based sensors suitable for biomolecule detection in harsh environments due to its high thermal stability and electrical insulation properties, but its non‐biodegradability limits its application. In contrast, biodegradable substrates offer environmentally friendly options for wearable health monitoring devices while maintaining the necessary mechanical strength and flexibility. The introduction of biodegradable substrates makes LIG‐based sensors more accessible and affordable, facilitating mass production for single‐use health monitoring applications such as sweat analysis.^[^ ^44^ ^]^

Laser light characteristics here mainly refer to the laser wavelength and modes of laser operation. On the one hand, varying wavelengths of laser light can produce distinct thermal effects and chemical reactions on the material surface, thus modulating the porosity, conductivity, and chemical functional group types of the LIG.^[^ ^45^, ^46^, ^47^ ^]^ Researchers observed a significant impact of laser wavelength on the critical laser fluence necessary for graphene conversion. Specifically, a fluence of ≈4.9 J cm^−2^ was required at a wavelength of 10.6 µm, whereas a lower fluence of ≈2.1 J cm^−2^ was sufficient at 9.3 µm.^[^ ^30^ ^]^ The ultrafast kinetics of lasing process are beneficial to the formation of unusual topologies.^[^ ^34^, ^48^, ^49^ ^]^ This affects its adsorption ability and reactivity toward biomolecules, thus directly affecting the sensitivity and specificity of the biosensor. Continuous short‐wavelength laser interaction with PI is generally dominated by photochemical reactions, while long‐wavelength lasers are dominated by photothermal reactions, which may lead to a larger heat‐affected zone.^[^ ^50^ ^]^ Therefore, short‐wavelength lasers are more suitable for the processing of more delicate structures at higher resolutions. On the other hand, modes of laser operation, including continuous and pulsed lasers, are also a key factor.^[^ ^51^ ^]^ Continuous lasers are suitable for efficient processing of large areas but may result in larger heat‐affected areas.^[^ ^52^ ^]^ Pulsed lasers offer high peak energy density and fine processing capabilities, allowing for smaller heat‐affected areas^[^ ^53^ ^]^ (such as “cold processing” with femtosecond laser^[^ ^54^ ^]^) and more detailed control of micro/nano‐structures.^[^ ^55^ ^]^ Continuous‐wave lasers are, therefore, suitable for sensor applications that require fast, large‐area processing, such as health monitoring devices for skin or clothing integration, while pulsed lasers are more suitable for the preparation of highly sensitive and specific biosensors.

The functionality of LIG is further enriched by the introduction of admixtures that not only improve the physical and chemical properties of LIG but also extend its effectiveness in specific application scenarios. For example, the introduction of admixtures such as graphene oxide (GO)^[^ ^56^ ^]^ or metal nanoparticles^[^ ^57^ ^]^ can significantly improve the sensitivity and selectivity of LIG sensors in detecting biomarkers at low concentrations. The addition of biorecognition molecules (such as antibodies or enzymes) can enable LIG‐based sensors to specialize in the detection of specific health‐related molecules, such as protein markers,^[^ ^58^ ^]^ and thus play an important role in early disease diagnosis and health status monitoring.

Overall, after a decade of continuous development and innovation, LIG technology has evolved from its initial discovery into a versatile technology with a wide range of applications in health monitoring. LIG‐based sensors' innovations in the selection of substrate materials, the optimization of laser wavelengths and types, as well as the precise introduction of admixtures not only improve the performance of the sensors but also broaden the range of their personal health management and healthcare applications. With future research, LIG‐based sensors are expected to play a more significant role in improving the quality of life and promoting scientific and technological innovations in the field of health.

## 3 LIG‐Based Sensing Mechanisms and Recent Advances in Health Monitoring

In this section, we systematically discuss the versatile sensing mechanisms of LIG‐based sensors, focusing on their recent advances in the field of health monitoring. LIGs, known for their unique electrical conductivity and structural properties, have garnered a great deal of attention in the field of sensor development, especially for applications in health‐related fields. This section aims to provide a comprehensive understanding of the multifunctional principles and performance of LIG‐based sensors. By examining extensive sensing mechanisms, the adaptability of LIG across distinct sensing modalities highlights its potential to provide comprehensive health monitoring solutions. Each subsection provides an in‐depth focus on a particular aspect of LIG sensing, illustrating how these mechanisms have been utilized to innovate in the field of health monitoring.

### 3.1 Electrochemistry

The advantages of LIG as an electrochemical sensor stem from its unique physical and chemical properties. Firstly, the excellent electrical conductivity and controllable 3D graphene structure of LIG are conducive to the sensitivity and response speed of electrochemical sensors.^[^ ^59^ ^]^ These enhancements provide a reliable basis for achieving rapid and accurate detection of weak changes in physiological parameters. Secondly, the mechanical strength and stability of LIG enable it to maintain high performance in complex environments.^[^ ^60^ ^]^ In addition, the abundant functional groups and defect sites on the surface of LIG provide it with excellent biocompatibility and surface activity.^[^ ^61^ ^]^ These properties serve as an excellent platform for efficient adsorption and sensing of biomolecules. The functional groups on the surface of LIG also allow it to be flexibly modified with biorecognition molecules, such as enzymes^[^ ^62^ ^]^ and antibodies,^[^ ^63^ ^]^ to achieve highly selective detection of specific biomolecules. Combining these advantages, LIG demonstrates excellent performance in electrochemical sensing and provides a solution for efficient and sensitive monitoring of physiological parameters, making it an ideal material for human health assessment. Therefore, LIG‐based electrochemical sensing showcases remarkable versatility in health monitoring. From the precise detection of biomolecules like uric acid and tyrosine to their contribution to the diagnostic landscape, including the detection of cortisol levels and even combating the challenges posed by COVID‐19, LIG sensors have emerged as indispensable tools.

**Figure 2:**
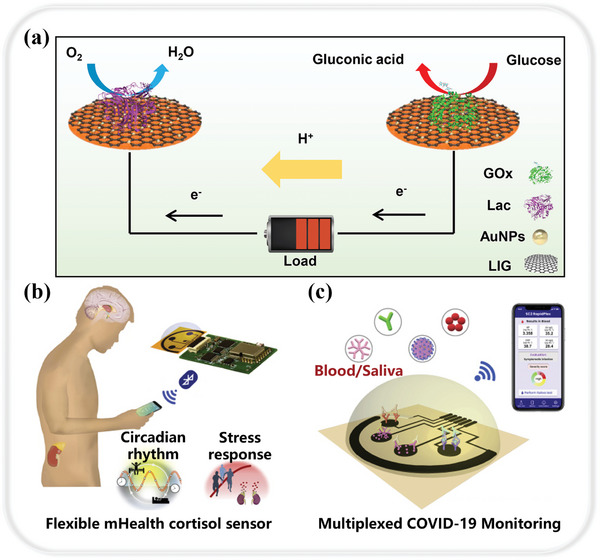
LIG‐based electrochemical sensing. a) the working principle of the proposed LIG‐based transient glucose enzymatic cells. Reproduced with permission.^[^ ^64^ ^]^ Copyright 2022, American Chemical Society. b) Schematic of an integrated wireless LIG‐based mobile health sensing device for noninvasive monitoring of the stress hormone cortisol. Reproduced with permission.^[^ ^69^ ^]^ Copyright 2020, Elsevier. c) LIG‐based immune‐sensor platform for COVID‐19 detection in blood and saliva. Reproduced with permission.^[^ ^70^ ^]^ Copyright 2020, Elsevier.

LIG‐based implantable medical electronics perform a vital function in clinical medicine. However, liquid electrolyte leakage from the batteries in the devices can cause damage to the surrounding tissues. Moreover, the batteries need to be replaced when depleted or malfunctioned, which may cause harm to the patients. Huang et al. investigated transient glucose enzymatic biofuel cells based on composite electrodes of LIG and gold nanoparticles, providing a new energy solution for future transient electronics and implantable medical devices.^[^ ^64^ ^]^ The working principle of the proposed LIG‐based transient glucose enzyme cell is shown in **Figure** **2**a. It not only improves the open‐circuit potential (0.77 V) and maximum power density (483.1 µW cm^−2^) of the bio‐battery, but also has a fast response time to reach the maximum open‐circuit potential within 1 minute and an in vitro lifetime of >28 days. With excellent biocompatibility and degradability, the cells are more suitable for implantable medical electronic devices and can be diversified according to different needs, which is promising for applications in supplying energy to microelectronic devices in the biomedical field.

Many chemicals excreted by the body through sweat can reveal health conditions, such as uric acid and tyrosine, both of which are key indicators for assessing health.^[^ ^65^, ^66^ ^]^ The level of uric acid, a waste product produced during metabolism, often reflects the state of kidney function or the presence of gout issues. Tyrosine, a basic amino acid, is not only a precursor to neurotransmitters such as dopamine, epinephrine, and norepinephrine but also an important component of thyroid hormones. Therefore, changes in tyrosine concentrations may indicate the health of the nervous system and the thyroid gland. Although these indicators are crucial for health monitoring, most wearable sweat sensors currently are only capable of detecting a few electrolytes and metabolites.^[^ ^67^ ^]^ Moreover, these sensors lack accurate measurements for components such as uric acid and tyrosine, which are not present at high levels. In this context, the wearable sweat sensor reported by Yang et al. demonstrates remarkable innovation in the field of electrochemical sensing.^[^ ^68^ ^]^ By combining a LIG‐based electrochemical sensor with microfluidics, the efficient processing of the sensing and microfluidic modules was achieved by utilizing a CO_2_ laser. Collecting sweat from the body surface through microfluidic channels reduces evaporation from the skin while maximizing the cleanliness and contamination‐free nature of the sweat. Compared with commercial glassy carbon and gold electrodes, LIG‐based sweat sensors have superior electrochemical performance. Featuring high temporal resolution and low contamination of sweat collection, the sensor is able to accurately monitor low concentrations of uric acid and tyrosine (0.74 and 3.60 µm, respectively), realizing a fast and accurate in situ detection of uric acid and tyrosine in human sweat and providing a new solution for monitoring cardiovascular diseases, metabolic disorders, and other conditions. In addition, this research demonstrated a substantial correlation, with a coefficient of 0.864, between concentrations of uric acid in sweat and serum, thereby indicating the prospective application of sweat uric acid as a biomarker in the management of gout.

Monitoring cortisol levels in sweat is equally crucial for human health performance analysis. With the accelerated pace of life, there is a general gradual increase in psychological and physiological stress, leading to an increased risk of mental health problems and chronic diseases.^[^ ^71^ ^]^ The quantification of stress via biochemical markers has garnered significant attention, with cortisol emerging as a paramount biomarker due to its pivotal role in stress response.^[^ ^72^, ^73^, ^74^ ^]^ However, the current main methods for monitoring stress levels include the use of highly subjective questionnaires and invasive blood sampling, emphasizing the need for more objective and practical approaches. To address this gap, Torrente et al. introduced an innovative approach to cortisol measurement, leveraging the advanced technologies in LIG‐based electrochemical sensing and mobile health systems (Figure 2b).^[^ ^69^ ^]^ This study delineates the development and application of a non‐invasive, real‐time method for monitoring cortisol levels in human sweat, a frontier in stress physiology and mental health monitoring. A flexible LIG‐based sensor array within an integrated wireless mobile health device was utilized, combining the sensitivity and specificity of LIG with competitive immune‐sensing to detect cortisol in human sweat and saliva. This system successfully monitored the diurnal variation of cortisol in human sweat and the response to acute stress stimuli. It can accurately quantify cortisol levels within 1 minute with high sensitivity (detection limit of 0.08 ng mL^−1^), providing a new perspective for non‐invasive, dynamic stress monitoring. The sensor's performance was evaluated against traditional methods, revealing a significant enhancement in sensitivity. The LIG sensor exhibited a nearly two‐fold and six‐fold reduction in current density compared with glassy carbon electrodes and screen‐printed carbon electrodes, respectively, when detecting changes in cortisol concentration from 0.0 to 1.0 ng mL^−1^. This superior performance is further corroborated by the high correlation coefficient (0.973) with the results of enzyme‐linked immunosorbent assay, the gold standard for cortisol quantification. These results not only endorse the sensor's efficacy but also highlight its potential as a reliable tool for cortisol monitoring.

In terms of electrochemical detection of specific biomarkers of COVID‐19, achieving early and ultrasensitive infection identification is critical to preventing the wide spread of COVID‐19.^[^ ^75^ ^]^ Challenges facing the public health system include effective identification of infected individuals, rapid response, and development of effective testing and tracking measures. Torrente et al. developed the SARS‐CoV‐2 RapidPlex, a multiplex electrochemical LIG‐based platform that enables sensitive and express simultaneous quantification of COVID‐19‐specific biomarkers in blood and saliva (Figure 2c), including the SARS‐CoV‐2 nucleocapsid protein, immunoglobulins specific for the SARS‐CoV‐2 spiking protein (IgM and IgG) and C‐reactive protein (CRP).^[^ ^70^ ^]^ The results of each bio‐indicator can be transmitted wirelessly to a cell phone with a detailed assessment (healthy, pre‐infected, infected but asymptomatic, infected and symptomatic, recently recovered, long‐term recovered, inflammation/infection not due to COVID‐19). The multiplex functionality of the SARS‐CoV‐2 RapidPlex was evaluated in serum samples from COVID‐19 RT‐PCR‐positive subjects, with significant positive readings for all targets obtained with only 1 min of incubation time. The signal‐to‐blank ratios for four biomarkers in serum and saliva samples from RT‐PCR‐positive COVID‐19 patients were remarkably higher (10.53, 11.62, 10.67, 12.39, and 2.81, 3.24, 1.62, 1.76, respectively) than those of RT‐PCR‐negative subjects. These specific biomarker test results demonstrated the utility of the LIG‐based electrochemical sensor for COVID‐19 monitoring. The platform, due to its simplicity and rapid assay time gives it great potential as a rapid diagnostic tool in the field.

Based on the above literature analysis, LIG‐based electrochemical sensing has proven to be a game‐changer in the realm of health monitoring. LIG's unique physical and chemical attributes, particularly its excellent electrical conductivity, controllable 3D structure, and surface rich in functional groups, have elevated its potential as a versatile and sensitive material for sensor applications. These qualities enable LIG‐based sensors to achieve rapid and accurate detection of various physiological parameters, ensuring high performance even in complex environments. Additionally, the biocompatibility and surface activity of LIG allows for the effective adsorption and selective sensing of biomolecules, further enhancing its utility in health assessments. The wide range of applications of LIG sensors, from monitoring essential biomarkers in bodily fluids such as sweat to their role in implantable medical electronics, demonstrates their significance in modern healthcare. These sensors' ability to detect critical health indicators non‐invasively and in real‐time addresses the growing need for practical, objective methods in health monitoring. The versatility and efficacy of LIG‐based electrochemical sensing in tracking physiological changes and stress levels, and even aiding in the fight against global health challenges like COVID‐19, highlight its indispensable role in advancing diagnostic technologies and personalized health management.

### 3.2 Potentiometry

Potentiometry, as a specific electrochemical sensing technique, focuses on the monitoring of physiological parameters by measuring the potential difference between the working electrode and the reference electrode.^[^ ^76^ ^]^ Of particular interest are the applications of LIG‐based potentiometric sensing in the monitoring of specific electrolyte concentrations crucial for human physiological balance, such as Na^+^ and K^+^ in body fluids, and their potential use in medical applications such as electrocardiography.^[^ ^77^ ^]^ These ions play a vital physiological role in keeping the heart functioning properly. Na^+^ is pivotal in maintaining cellular homeostasis, nerve impulse transmission, and fluid balance, while K^+^ is involved in regulating the contraction and diastole of the heart muscle and is essential for the maintenance of a regular heartbeat.^[^ ^78^ ^]^ Therefore, monitoring changes in the concentration of these ions by potentiometry can provide significant information about the functional state of the heart, offering an innovative approach to the timely monitoring of cardiovascular health. Furthermore, the high sensitivity and selectivity of LIG‐based potentiometric sensing offers new opportunities to achieve precise monitoring of these specific physiological parameters. This subsection discusses in depth the advancements of LIG‐based potentiometric sensors, mainly focusing on their applications in analyzing sweat and urine, crucial biofluids in health diagnostics.

As we discussed in the previous subsection, sweat analysis can be easily conducted on the skin, unlike blood monitoring, providing valuable data for personal health care and disease diagnosis. One of the pivotal studies in this area developed disposable devices capable of detecting Na^+^ in sweat using UV‐Ozone irradiated potentiometric LIG‐based electrodes.^[^ ^79^ ^]^ This novel approach enhanced sensor performance, with a sensitivity of 60.2 ± 0.9 mV dec^−1^ and a lower detection limit of 1 × 10^−6^ m. The response time of ≈1 min highlights its potential as a fast, non‐invasive sweat‐sensing wearable device. The significant improvement in ion sensing performance is attributed to the increased electroactive surface area and porosity of the LIG, which improves the sensitivity and reliability of the electrode in applications. These enhancements stem from the use of UV ozone irradiation along with improved attachment of the ion‐selective membrane to the LIG electrode. These properties are critical for the development of wearable health monitoring devices that can provide continuous, real‐time data for health assessment.

To expand the scope of sweat analysis, another study introduced a multifunctional LIG‐based sensor capable of simultaneously monitoring Na^+^ and K^+^ ion concentrations alongside physical strain.^[^ ^80^ ^]^ This wearable multifunctional porous LIG‐based sensor, fabricated on PDMS and lignin composite substrates through laser irradiation, exhibited high sensitivities of 63.6 mV dec^−1^ for Na^+^ and 59.2 mV dec^−1^ for K^+^. The outcomes of this study closely align with Nernstian principles, as evidenced by the high R^2^ values of 0.99988 and 0.99855, respectively. Additionally, the sensor demonstrates remarkable cyclic stability, enduring up to 5000 cycles with a Gauge Factor of ≈20. The straightforward manufacturing process of this versatile and economical sensor platform effectively captures real‐time fluctuations in Na^+^ and K^+^ levels in sweat, as well as the strain caused by human movement. This multifunctional sensor exemplifies the potential of LIG‐based technology in creating devices that can track various health indicators, providing a holistic view of an individual's physiological state.

**Figure 3:**
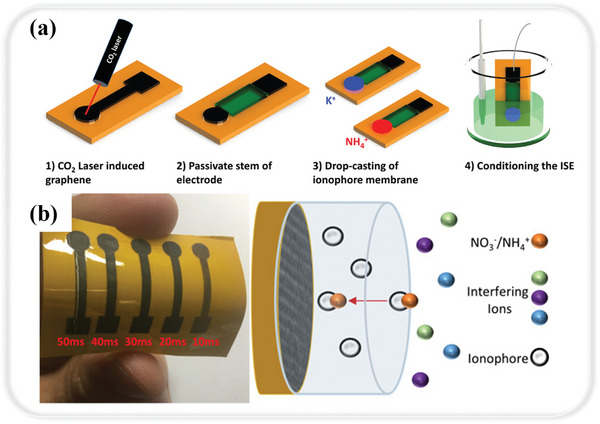
LIG‐based potentiometric sensing. a) Schematic representation of the LIG ISEs fabrication process. Reproduced with permission.^[^ ^81^ ^]^ Copyright 2020, John Wiley and Sons. b) The prepared LIG‐based electrodes and their ion‐selective sensing ion sensing mechanism. Reproduced with permission.^[^ ^82^ ^]^ Copyright 2018, American Chemical Society.

Further expanding the scope of LIG‐based biosensors to urine analysis, Kucherenko et al. developed LIG‐based ion‐selective electrodes (ISEs) for sensing K^+^ and NH_4_^+^ in urine for monitoring hydration in patients (**Figure** **3**a).^[^ ^81^ ^]^ The potentiometric sensor was integrated via LIG with ion‐selective membranes for NH_4_^+^ and K^+^. These electrodes exhibit remarkable stability, maintaining consistent signal strength over three months of storage. The NH_4_^+^ retained 86% of its sensitivity, while the K^+^ preserved 100% sensitivity, across a broad pH range from 3.5 to 9.0. The electrodes showcased near Nernstian sensitivities, recording 51 mV dec^−1^ for NH_4_^+^ and 53 mV dec^−1^ for K^+^, with detection thresholds at 30 × 10^−6^ m for NH_4_^+^ and 100 × 10^−6^ m for K^+^. Their rapid response time of 30 s makes them comparable to traditional hydration monitoring methods such as specific gravity measurements. The efficacy of these ISEs was further validated through the analysis of urine samples from both a young patient (aged 22) and an older patient (aged 73), successfully determining different levels of NH_4_^+^ and K^+^. This analysis confirmed the hydration status of the patients, illustrating the ISEs’ practicality and reliability in diverse age groups and conditions.

In addition to the direct monitoring of human indicators, environmental factors also have a significant indirect impact on human health. In agricultural practices, the overutilization of nitrogen‐rich fertilizers has been a major contributor to soil deterioration and subsequent contamination of aquatic ecosystems.^[^ ^83^ ^]^ Such activities often lead to the excessive accumulation of nitrates in water bodies, fostering harmful algal growth and posing significant health hazards to human populations. Addressing this issue, Garland et al. developed a LIG‐based potentiometric ion‐selective sensing of essential plant nutrients with a cost‐effective UV laser, namely NH_4_^+^ and NO_3_^–^ in soil (Figure 3b).^[^ ^82^ ^]^ In optimizing LIG's electrochemical reactivity, the researchers strategically operated the laser at varying pulse widths from 10 to 50 milliseconds and observed that a specific setting of 20 milliseconds was particularly effective in creating LIG with a high sp^2^ carbon content of 77%. This optimized LIG was then utilized to construct electrodes, specifically tailored with distinct ionophores, resulting in the formation of unique solid contact ion‐selective electrodes for the respective nitrogen forms. The prepared LIG‐based ion‐selective electrodes exhibited near Nernstian sensitivities of 51.7 ± 7.8 mV dec^−1^ for NH_4_^+^ and −54.8 ± 2.5 mV dec^−1^ for NO_3_^–^. Their detection limits were also noteworthy, being 28.2 ± 25.0 µM for NH_4_^+^ and 20.6 ± 14.8 µM for NO_3_^–^, along with low long‐term drift rates (0.93 mV h^−1^ for NH_4_^+^ sensors and −5.3 µV h^−1^ for NO_3_^–^ sensors). In addition, they functioned effectively across a linear sensing range of 10^–5^ to 10^–1^ M. When applied to soil slurries, these sensors achieved substantial recovery rates, 96% for NH_4_^+^ and 95% for NO_3_^–^. This study not only highlights the effective performance of these sensors but also emphasizes their utility in soil health management. Their efficient fabrication process, avoiding the need for metallic nanoparticle enhancements, further underscores their potential for widespread application in diverse agricultural and environmental settings. For water quality analysis, Hjort et al. proposed the use of hydrophobic LIG for the preparation of a nitrate ISE.^[^ ^84^ ^]^ The sensor, with a contact angle of 135.5° (water), was fabricated by a dual laser process and has a Nernstian response (− 58.2 ± 4.2 mV dec^−1^) and a low detection limit (6.0 ± 1.4 µm). In a practical application scenario, this sensor was tested for its capability to analyze water samples, and its performance was benchmarked against a US standard analytical method. The results revealed that the sensor's results were statistically comparable to those obtained from the established method. Furthermore, the study extended to assess the long‐term stability and robustness of the sensor. Over five weeks, the sensors were submerged in surface water samples to monitor any potential degradation in performance. Remarkably, the sensors demonstrated consistent performance throughout this period, with no significant variations in both limits of detection and sensitivity. This finding underscores the sensor's reliability and potential for long‐term monitoring of surface water quality, offering an insight into the durability of such technologies in field conditions. The development of these potentiometric sensors signifies the expanding role of LIG in different fields, bridging the boundary between environmental science and health diagnostics.

The exploration of LIG in potentiometric sensing for health monitoring has demonstrated its versatility and efficiency in creating sensitive, accurate, and non‐invasive sensors. The advancements in LIG‐based sensors for sweat analysis, coupled with their applications in environmental monitoring, highlight the broad potential of this technology. The continued development and application of LIG in potentiometric sensing are poised to revolutionize the fields of healthcare and environmental science, offering new insights and tools for monitoring and understanding health and environmental conditions.

### 3.3 Thermoelectricity

Thermoelectric sensing is achieved by measuring the thermoelectric effect produced by a material under a temperature gradient.^[^ ^85^ ^]^ LIG exhibits excellent thermoelectric conductivity, and its thermoelectric properties induce charge separation, leading to a voltage difference when distinct regions of the material are exposed to varying temperature environments.^[^ ^86^ ^]^ By measuring the change of this voltage difference, the monitoring of temperature change can be realized, providing strong support for health monitoring. Meanwhile, the distinctive 3D graphene network structure of the LIG provides a good electronic conduction channel for thermoelectric sensing, which aids in the effective conversion of minor temperature fluctuations into measurable electrical signals, enabling sensitive temperature monitoring.^[^ ^87^ ^]^

In the field of flexible electronics, the production of LIG by irradiating common paper, known as paper‐LIG, marks a pivotal advancement.^[^ ^43^ ^]^ This approach aligns with the growing trend toward utilizing cost‐effective, sustainable materials in electronic device fabrication. The concept of paper‐LIG resonates with the shift in focus toward environmentally friendly technologies, highlighting the potential of using everyday materials in advanced applications. In the broader context of LIG research, the exploration of the effects of different laser parameters, such as the frequency and the beam scanning speed, has been crucial. These studies have helped in understanding the relationship between the processing conditions and the resulting material properties, such as structure, morphology, and electrical conductivity. Such insights are instrumental in tailoring LIG for specific applications, particularly in sensing technologies. Kulyk et al. delved into the fabrication of paper‐LIG temperature sensors, accomplished via a straightforward process: irradiating filter paper with a pulsed UV laser at 355 nm.^[^ ^88^ ^]^ This method is noteworthy for its preference toward photochemical reactions, offering a shallow penetration depth compared to traditional methods. In this study, a detailed analysis is presented of how various process parameters influence the transformation of cellulose fibers into LIG, examining aspects like morphology, structure, and chemical composition. This analysis uncovers unique transformational barriers and propagation behaviors, distinguishing UV laser irradiation from the more conventional CO_2_ laser approach. The resultant paper‐LIG material features a porous, electrically conductive network of fibers (5–10 nm few‐layer graphene flakes), leading to the development of temperature sensors with impressive sensitivities. These sensors are rigorously evaluated for their linearity, reproducibility, and response time, showcasing their efficacy. The sensors exhibited clear, stable, and reproducible responses, particularly at 20% RH, with minimal drift in resistance values. Linear regression analysis of the data further confirmed the sensors' high linearity, with coefficients of determination nearing or exceeding 0.99. Notably, the highest sensitivity was recorded at 50% RH during the temperature increase phase, reaching as high as −2.8 × 10^−3^ °C^−1^.

**Figure 4:**
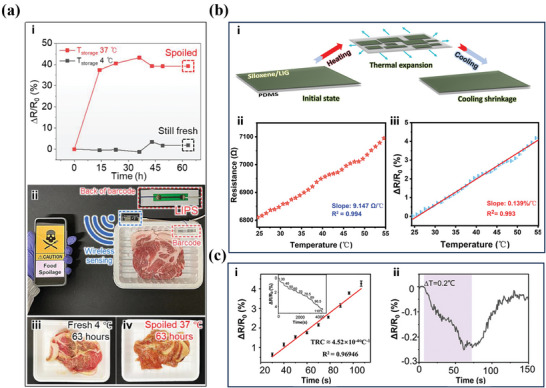
LIG‐based thermoelectric sensing. a) Real‐time wireless monitoring of the ambient temperature of raw pork. Reproduced with permission.^[^ ^92^ ^]^ Copyright 2022, Elsevier. b) Working mechanism and performance of the proposed LIG‐based temperature sensor. Reproduced with permission.^[^ ^93^ ^]^ Copyright 2022, John Wiley and Sons. c) Sensitivity testing and showcase of the sensor's capability to detect a minor temperature. Reproduced with permission.^[^ ^94^ ^]^ Copyright 2020, John Wiley and Sons.

The advancements in paper‐LIG, particularly in the development of temperature sensors with high sensitivity and linearity, demonstrate the material's potential for a broad range of applications. This adaptability is particularly relevant in the context of growing global concerns about food quality and safety. The necessity for vigilant monitoring of food quality and safety is underscored by the alarming statistics of food poisoning, which has impacted ≈600 million individuals in recent decades, tragically resulting in ≈420 000 deaths annually.^[^ ^89^ ^]^ A critical factor in mitigating this risk lies in the effective monitoring of food temperature. Most food products are prone to accelerated chemical decomposition when not stored at appropriate temperatures,^[^ ^90^ ^]^ which also affects their shelf‐life estimation. This reality propels the demand for innovative solutions to assess the condition of food items, particularly those packaged in paper‐based materials, without having to physically open and check for spoilage.^[^ ^91^ ^]^ In this context, the development of a smart, real‐time monitoring system becomes essential not only for consumer safety but also for enhancing the efficiency of storage and supply chains within the food industry. Traditional methods using LIG on PI films face limitations in green electronics due to PI's non‐biodegradable nature.^[^ ^24^ ^]^ To overcome this, Jung et al. introduced a new approach with their laser‐induced paper sensor (LIPS), a novel LIG/paper hybrid structure that facilitates wireless, real‐time monitoring of food status through its thermal and chemical sensing capabilities.^[^ ^92^ ^]^ The sensor is created by directly irradiating commercial paper of any type or structure, transforming the surface into LIPS characterized by suitable sheet resistance (105 Ω sq^−1^) and a porous structure. This allows for the efficient monitoring of crucial parameters like temperature, which is vital for detecting potential spoilage before consumption. The study found that optimized 532 nm continuous‐wave laser irradiation conditions on the paper substrate yielded a temperature coefficient of resistance change of 0.15% ˚C^−1^. Interestingly, the type of paper used does not affect LIPS's temperature sensing performance, as demonstrated in tests with paper cups and colored papers. The practicality of LIPS was verified through its successful integration into protein‐rich food packaging, where it adeptly monitored transient changes in food states, both refrigerated (≈4 ˚C) and warm (37 ˚C). For example, the LIPS‐based thermoelectric sensor was processed on the back side of the barcode of a raw pork safe, as shown in **Figure** **4**a. This affirms its utility in everyday scenarios for tracking food quality. However, it is worth noting that the response time of 51.5 s for LIPS temperature sensing may need to be further optimized for more immediate applications. Nonetheless, this innovative sensor stands as a significant advancement in the realm of food safety, offering a smart, eco‐friendly solution to a globally pressing issue.

The principle of varying thermal expansion rates among different materials, particularly evident in the behavior of the PDMS substrate, serves as a foundational concept in thermoelectric sensor technology. This phenomenon, where the expansion and contraction of PDMS lead to the formation of micro‐cracks, significantly affects the electrical resistance of the material.^[^ ^95^ ^]^ These micro‐cracks are not permanent and demonstrate reversibility, highlighting the material's resilience and capacity to adapt to temperature fluctuations.^[^ ^96^ ^]^ Based on this fundamental principle, a recent study reported the development of a siloxane/LIG/PDMS‐based temperature sensor (Figure 4b).^[^ ^93^ ^]^ This sensor stands out due to its distinctive electrode design, which mimics the intricate patterns of a butterfly's wings and is just 1 mm wide. This design is crucial for its function, as it incorporates a lengthy electrical path and significant initial resistance, key factors for enhancing temperature detection sensitivity. The sensor can reliably detect temperature changes in the range of 25 to 55 °C with a sensitivity of 0.139% °C^−1^ and a linear resistance response sensitivity of 9.147 Ω °C^−1^. The fusion of Siloxene and LIG on a PDMS base, through a novel C─O─Si bonding process, extends its application beyond mere temperature measurement to include human perspiration and skin temperature monitoring. This combination of flexibility, precision, and stability in the sensor's design and functionality marks a significant advancement in non‐invasive temperature monitoring technologies.

As for the precise adjustment of the porous structure of the LIG, this remains a difficulty when using normal carbon‐based polymers in lasering processes. To overcome this problem, researchers have explored the use of self‐assembled block copolymers in direct laser writing, and recent studies have shown that this approach can help in the fabrication of LIGs with hierarchical porous structures.^[^ ^97^, ^98^, ^99^ ^]^ Vanadium oxide (VO_x_), a transition metal oxide, is noted for its exceptional characteristics, such as chemical and thermal stability, and superior thermoelectric properties. Despite these advantages, the implementation of VO_x_ and its composites has been hindered by intricate synthesis methods, such as hydrothermal,^[^ ^100^ ^]^ electrospinning^[^ ^101^ ^]^ and spray pyrolysis^[^ ^102^ ^]^ processes. Recent reports on the direct synthesis of VOx with lasers offer motivation and possibilities for the simultaneous synthesis of laser‐induced VOx and graphene.^[^ ^102^, ^103^ ^]^ Yang et al. have reported a novel one‐step method using laser direct writing to synthesize VOx‐doped porous LIG composites.^[^ ^94^ ^]^ This process involves laser engraving block copolymers (BCPs) doped with a V_5_S_8_ precursor. Unlike typical carbon‐based polymers that utilize a single monomer, this method employs Pluronic F127‐resols with an adjustable mass ratio of Pluronic F127 copolymer to resols mixture in ethanol. This enables a bottom‐up self‐assembly process that precisely controls mesostructures and pore size distribution. The Pluronic F127‐resols film facilitates the modulation of the porous structure through the mass ratio of BCP to resins, allowing VOx particles to anchor onto the porous LIG without clustering.^[^ ^104^ ^]^ A significant outcome of this study is the formation of a heterojunction at the VOx/LIG interface, which substantially elevates the sensor's performance in temperature sensing. The resulting sensor demonstrates a wide temperature detection range with a sensitivity of 4.52 × 10^−4^% °C^−1^ and a low detection limit of 0.2 °C, as shown in Figure 4c. Additionally, it shows remarkable repeatability across heating‐cooling cycles, proving effective in monitoring both subtle and significant temperature shifts in practical scenarios. The sensor's practical applications were demonstrated, including monitoring the temperature of formula milk in a bottle for infants and simulating fever conditions, with a significantly faster response time than traditional commercial mercury thermometers.^[^ ^105^ ^]^ These findings validate the high sensitivity, broad detection range, rapid response, and reliability of the VOx‐doped LIG temperature sensor, making it highly suitable for dynamic temperature monitoring applications. When integrated with data processing and wireless transmission modules, this sensor paves the way for a comprehensive remote environmental monitoring system, capable of wirelessly tracking temperatures for human health monitoring purposes.

High‐precision temperature monitoring is essential across various sectors, with a particular emphasis on its importance in biomedicine. In specific medical scenarios such as cardiac and neurosurgery, the meticulous control and monitoring of body temperature during intraoperative and postoperative phases play a pivotal role in mitigating complications. The challenge in this field arises from the introduction of a temperature sensor into a thermal environment, which can cause perturbations leading to measurement inaccuracies. Such inaccuracies are not trivial, as they can have significant implications.^[^ ^106^ ^]^ Traditional solutions, like the zero‐flux thermometer, offer precision but are hindered by lengthy equilibration times, sometimes up to 5 min.^[^ ^107^ ^]^ The advent of transformation thermotics theory^[^ ^108^ ^]^ has been a game‐changer, catalyzing the creation of innovative thermal manipulation meta‐devices. These devices, including thermal concentrators,^[^ ^109^ ^]^ cloaks,^[^ ^110^ ^]^ encodings,^[^ ^111^ ^]^ and camouflages,^[^ ^112^ ^]^ aim to revolutionize temperature measurement. Among them, the thermal invisibility sensor stands out for its potential to measure temperatures accurately without disturbing the original thermal field.^[^ ^113^ ^]^ This is achieved by encasing the sensor in one or two layers of meta‐structure, allowing heat to flow undisturbed and ensuring both accurate measurement and thermal invisibility.^[^ ^114^ ^]^ However, the practical application of such thermal meta‐devices is often constrained by the need for certain materials and precise thermal conductivity distribution,^[^ ^115^ ^]^ which can be challenging to implement. Addressing these challenges, Hou et al. introduced a meta‐shell encircled LIG‐based temperature sensor specifically designed for high‐precision temperature monitoring.^[^ ^116^ ^]^ The theoretical aspects of the sensor's thermal monitoring capabilities highlight its ability to nullify thermal perturbations caused by sensor‐background mismatches, thereby ensuring more accurate readings. Experimentally, the sensor's performance was validated using UV laser scanning, a technique employed to fine‐tune the thermal conductivity of LIG films through varying fabrication parameters. This novel sensor exhibited a significantly reduced thermal deviation of just 0.21 K compared to 1.55 K by a bare sensor, indicating its superior accuracy. Additionally, the research confirmed that this LIG‐based thermoelectric sensor maintains its high accuracy even in extreme temperatures exceeding 410 K, demonstrating its robustness and reliability through experimental validation of its stability and repeatability. Notably, the sensor displayed a rapid response time of merely 1.15 s and proved capable of delivering precise temperature measurements even under significant curvature (0.4 m^−1^) and showing no sensitivity to humidity or vibration. These attributes underscore its versatility in various challenging environments. The LIG‐based thermal meta‐shell encircled sensor presents several compelling advantages: adjustable thermal conductivity, high‐resolution patterning, exceptional measurement accuracy, ease of processing, and flexibility. These characteristics render it particularly suited for applications requiring rapid and precise miniaturized temperature measurements. This study not only marks a significant stride in the realm of temperature sensing technology but also opens avenues for broader applications where precise thermal regulation and measurement are crucial. The high measurement accuracy, tunable thermal conductivity, and high‐resolution patterning of LIG‐based thermal sensors render it particularly suited for applications requiring rapid and precise miniaturized temperature measurements. This research not only marks a significant stride in the realm of temperature sensing technology but also opens avenues for broader applications where precise thermal regulation and measurement are crucial.

In addition to temperature information, LIG‐based thermoelectric sensing technology is expected to monitor blood flow. Since blood carries away body temperature as it flows, the rate of blood flow and blood circulation can be inferred by monitoring subtle temperature changes. This is potentially important for the assessment of cardiovascular health and the early diagnosis of some diseases. Overall, LIG‐based thermoelectric sensing provides not only accurate temperature information but holds the promise of indirect access to key parameters related to blood flow, providing multifaceted information to support comprehensive human health monitoring.

### 3.4 Piezoresistivity

The core of piezoresistive sensing is to utilize the property of material resistance change under external force.^[^ ^117^ ^]^ When external pressure is applied to a pressure‐sensitive material, the lattice structure of the material deforms. This deformation leads to a change in carrier mobility, causing a measurable change in resistance. By constructing the relationship between the pressure source and the resistance, the monitoring of relevant parameters can be realized.^[^ ^118^ ^]^ In terms of human physiological indicators, piezoresistive sensing is able to realize real‐time monitoring of key indicators such as respiratory conditions, sound, and blinking. Carbon materials such as carbon nanotubes (CNTs), graphene, and their composites are widely used in airflow sensors because of their high stability, lightweight, and high electron mobility. However, CNT‐based devices are more expensive to prepare, more difficult to produce on a large scale, and less biocompatible.^[^ ^119^ ^]^ Compared with CNTs, LIG is not only simpler and cost‐effective to prepare, but also performs better in terms of biocompatibility and enables safer contact with human tissues, making it more suitable for health monitoring.

For monitoring breathing conditions, LIG can be used as an airflow sensor. When the body's exhaled airflow comes into contact with the LIG, the airflow pressure affects the resistance of the LIG. By continuously monitoring the changes in LIG resistance, information such as the frequency, depth, and pattern of breathing can be obtained. The combined information can be used to assess an individual's respiratory health, such as asthma and chronic obstructive pulmonary disease. Stanford et al. reported a LIG‐based gas sensor, whose sensing principle is based on the heat exchange between heated graphene and gas, which requires a continuous heat supply and high energy consumption.^[^ ^120^ ^]^ At the same time, the response time of the sensor is slow, requiring up to ≈30 s of response time for stable signal acquisition. Under the piezoresistive sensing principle, recent studies have been conducted to improve the sensing response speed by modulating the 3D structure of LIG.^[^ ^121^, ^122^ ^]^ Reasonable tuning of the three‐dimensional structure and understanding the effect of the structure on signal transmission are important for designing and optimizing airflow sensors. The intricate structures found in nature have long been a source of inspiration for biomimetic research, particularly in overcoming the constraints of traditional synthesis technologies. A prime example of this is the structure of butterfly wings, which feature scales with complex periodic architectures. This natural design has captivated materials scientists, leading to the widespread study and applications of bionic functional materials. These applications span various fields, including the development of medical devices,^[^ ^123^ ^]^ sensors for volatile organic compounds,^[^ ^124^ ^]^ and photothermal materials.^[^ ^125^ ^]^ Focusing on the sensory aspects of Lepidopterans, specifically butterflies and moths, the sensory bristles and scales located along their wing margins and veins are of particular interest.^[^ ^126^ ^]^ These structures play a crucial role in regulating wing movement, aiding these creatures in their migration and orientation.^[^ ^127^, ^128^ ^]^

**Figure 5:**
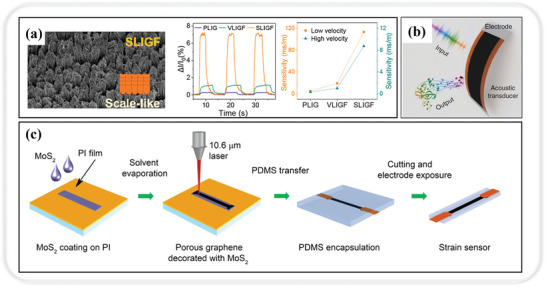
LIG‐based piezoresistive sensing. a) SEM image of SLIGF with fast response speed and high sensitivity. Reproduced with permission.^[^ ^129^ ^]^ Copyright 2023, American Chemical Society. b) The proposed LIG with the dual capability of both emitting and detecting sound within a single device. Reproduced under terms of the CC‐BY license.^[^ ^133^ ^]^ Copyright 2017, The Authors, published by Springer Nature. c) Schematic diagram of the fabrication process of MoS_2_‐modified LIG strain sensors. Reproduced with permission.^[^ ^134^ ^]^ Copyright 2019, American Chemical Society.

Our recent study addressed the issue of prolonged response times in LIG‐based airflow sensors by proposing bionic structure modulation of graphene. This approach involves laser and post‐processing techniques, optimizing laser‐induced conditions, and delving into the influence mechanism of how various multilevel bionic structures affect airflow signal generation and transmission.^[^ ^129^ ^]^ Bionic structural engineering modifies the deformation behavior of LIGs under pressure, with the synchronous propagation of Lepidoptera scale‐like suspended LIG fiber (SLIGF) structures being the most favorable to airflow sensing, as shown in **Figure** **5**a. The SLIGF airflow sensor demonstrates outstanding performance in various key metrics, positioning it as a remarkable advancement in the field of airflow sensing technology. With an impressively short average response time of just 0.5 s, it ranks among the fastest when compared with state‐of‐the‐art piezoresistive airflow sensors.^[^ ^130^, ^131^, ^132^ ^]^ Moreover, the SLIGF sensor with high sensitivity (0.11 s m^−1^) highlights its ability to detect subtle changes in airflow patterns with remarkable precision. In addition to its responsiveness and sensitivity, the SLIGF sensor establishes a remarkable record‐low detection threshold (0.0023 m s^−1^). This represents a significant breakthrough, as it allows for the detection of even the slightest airflow movements, making it suitable for a wide range of applications that demand high‐precision airflow measurements. Beyond its exceptional technical specifications, our study also demonstrates the practical utility of SLIGF airflow sensors in healthcare. Overall, this study not only contributes to the advancement of airflow sensing technology but also lays the foundation for the development of next‐generation sensors in areas such as water flow, sound, and motion detection.

For sound sensing, the propagation of sound impacts the resistance of the LIG. Since the sound wave exerts pressure on the medium during propagation, it results in a change in the resistance of the LIG. By monitoring the change in the resistance of the LIG, parameters such as the frequency and amplitude of the sound can be indirectly measured to assess the condition of the throat. Due to various health conditions and unforeseen accidents, a significant portion of the global population faces the challenge of communication impairment, rendering them unable to articulate their thoughts and feelings through conventional language.^[^ ^133^ ^]^ Remarkably, many individuals with speech disabilities still possess the ability to emit sounds, albeit often in the form of nonspecific vocalizations such as coughing, humming, or screaming, which lack clear intelligibility to others.^[^ ^135^ ^]^ It is imperative to address this issue by developing an accessible artificial vocal apparatus capable of converting ambiguous vocalizations into articulate and comprehensible language. This necessitates the artificial vocal apparatus to possess dual functionalities, the capability to both perceive and produce sound. Nevertheless, existing acoustic transducers designed for such applications typically operate within a limited bandwidth, often confined to ultrasonic frequencies.^[^ ^136^ ^]^ Furthermore, conventional sound generation and detection mechanisms tend to be discrete and isolated from one another, falling short of addressing the spectrum of human hearing effectively. To prepare miniature sound sensors and seamlessly integrate them into wearable e‐devices, Tao et al. introduced an innovative solution in the form of a smart LIG‐based artificial throat.^[^ ^133^ ^]^ This groundbreaking device not only exhibits the capacity to produce sound but also excels in sound perception, uniting both functions within a single apparatus (Figure 5b). In its role as a sound detector, the LIG throat exhibits distinctive responses to various types of sounds and throat vibrations, showcasing its versatility in sound perception. More significantly, this artificial throat holds the potential to change the lives of people with communication disorders. It is proficient at detecting and converting simple throat vibrations, each with varying intensities and frequencies, into manageable and intelligible auditory outputs.

For the monitoring of blinking movements, the LIG is placed around the eye or on the eyelid and senses the eye blinking by monitoring the pressure changes. When the eyelids are closed or opened, pressure is exerted on the surrounding medium, resulting in a change in the resistance of the LIG, which translates into information such as the frequency, speed, and strength of the blink.^[^ ^137^ ^]^ This approach can be used in studies such as assessing eye health or fatigue levels. Chhetry et al. introduced a highly responsive and dependable strain sensor based on piezoresistive sensing.^[^ ^134^ ^]^ This sensor is engineered through a one‐step process involving the carbonization of a polyimide film coated with MoS_2_, resulting in the preparation of MoS_2_‐modified LIG (Figure 5c). The outcome is a 3D porous LIG nanosheets adorned with MoS_2_ nanoparticles, which imparts remarkable stability to its electrical properties. This stability ensures a consistent and reliable output even over prolonged cycles of strain and release. The exceptional performance of this sensor extends its utility to the detection of subtle movements, such as eye blinking. The ability to capture instantaneous changes in resistance during blinking and relaxation, which can be monitored up to 22 cycles in 30 s, underscores its potential for health monitoring and integration into human‐machine interfaces. A compelling instance of the integration is the development of an innovative smart wireless human‐machine interface system based on LIG‐patterned arrays.^[^ ^138^ ^]^ This system enables the wireless manipulation of personal electronic devices through a simple touch. Interaction with the LIG‐patterned numeric touch panel generates raw output voltage signals from the system, which are then converted into square wave signals by a processing circuit. These signals are interpreted by a microcontroller unit, which counts peak numbers and issues command signals to a wireless module, facilitating communication with personal electronic devices. As evidence of the broad applicability and adaptability of LIG in enhancing interactive capabilities, a growing body of research has been dedicated to combining LIG‐based sensors with machine learning techniques, paving the way for significant improvements in intelligent health monitoring and interactive technologies.^[^ ^139^, ^140^ ^]^ Han et al. designed smart gloves that utilize LIG‐based pressure sensors combined with deep learning algorithms.^[^ ^141^ ^]^ These gloves are capable of achieving object classification recognition with 92% accuracy, showcasing how LIG technology can be extended beyond traditional sensing applications to include sophisticated analytical tasks. Following a similar innovative trajectory, Chen et al. have demonstrated that by integrating an artificial synapse with a 5 × 5 LIG‐based tactile sensing array.^[^ ^142^ ^]^ The artificial sensory neural network system is adept at recognizing various handwritten letters, achieving a remarkable accuracy of 94.44%. This advancement emphasizes the versatility and effectiveness of LIG‐based systems in deciphering complex patterns. These works illustrate the potential of LIG‐based sensors when combined with machine learning, to not only monitor and process complex biological signals but also to enable more intelligent and efficient health monitoring and human‐machine interaction applications.

In summary, piezoresistive LIG‐based sensors have demonstrated significant advantages in terms of biocompatibility, ease of preparation, and potential for application in the monitoring of multiple human physiological indicators. This non‐invasive, painless, and harmless monitoring method is of great significance in the fields of disease diagnosis, health management, and exercise science. It can help doctors identify potential health problems promptly and provide personalized treatment plans for patients. In addition, it also helps athletes and fitness enthusiasts to understand their physical condition, adjust training programs, and improve sports performance. Importantly, the integration of LIG‐based sensors with machine learning algorithms holds the promise of transforming health monitoring, offering predictive, and personalized health insights that were previously unattainable. With the continuous progress and optimization of technology, LIG‐based piezoresistive sensing, augmented by machine learning, will play an increasingly critical role in the monitoring of human physiological indicators, bringing more convenience and protection to our health and life.

### 3.5 Capacitance

LIG‐based capacitive sensors have a broad range of applications in the field of health monitoring,^[^ ^143^ ^]^ of which respiratory humidity monitoring is a typical application. The principles and the advantages of LIG as a sensing material are described below as well as recent research advances.

Firstly, respiratory humidity monitoring exploits the sensitivity of LIG‐based capacitive sensors to humidity. When the human body breathes out gas containing water vapor, its humidity is closely related to the condition of the respiratory tract. LIG sensors are able to sense this change in humidity and convert it into a change in capacitance.^[^ ^144^ ^]^ By measuring the change in capacitance, the respiratory humidity can be accurately monitored and thus the health of the respiratory tract can be assessed. The advantages of LIG in such monitoring include its excellent flexibility and sensitivity. LIG material has excellent flexibility and can adapt to a variety of curved and complexly shaped surfaces, fitting snugly to the human skin to ensure accurate monitoring of respiratory humidity. At the same time, LIG's high electrical conductivity and large specific surface area make it ultra‐sensitive to changes in humidity, enabling it to capture small changes in humidity and provide accurate respiratory humidity data.

**Figure 6:**
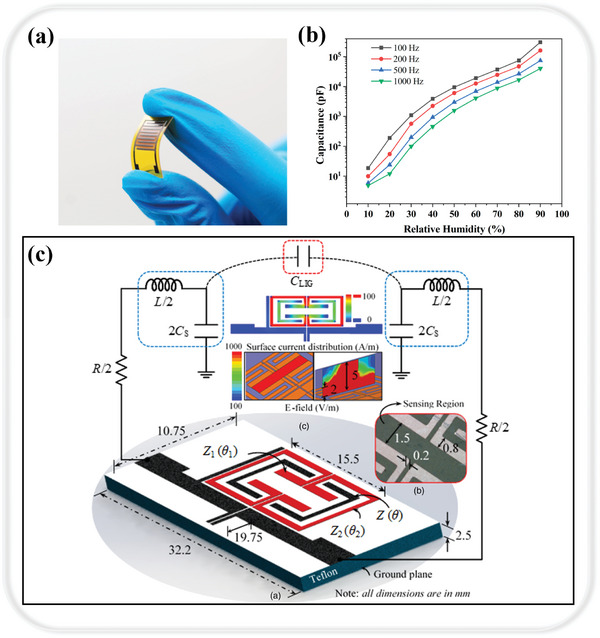
LIG‐based capacitive sensing. a) The developed flexible capacitive humidity sensors. Reproduced with permission.^[^ ^146^ ^]^ Copyright 2020, Elsevier. b) Relative humidity of the sensor in an optimal GO dosage range in relation to its capacitance response. Reproduced under terms of the CC‐BY license.^[^ ^147^ ^]^ Copyright 2023, The Authors, published by MDPI. c) The overall layout of the microwave‐LIG sensor. Reproduced with permission.^[^ ^148^ ^]^ Copyright 2019, IOP Publishing.

Recently, graphene oxide has been selected as an additive to assist in improving the humidity sensing performance of LIG due to its unique two‐dimensional structure and super permeability to water molecules.^[^ ^145^ ^]^ Researchers have developed an innovative and efficient method for large‐scale production of high‐performance flexible capacitive humidity sensors (**Figure** **6**a).^[^ ^146^ ^]^ This technique generates graphene‐interdigitated electrodes directly on PI films by laser induction (LIG‐IDE), marking a departure from the more complex and expensive processes traditionally used for IDE preparation. Enhancing the performance of these humidity sensors, the team incorporated GO into LIG. The resulting LIG/GO‐based capacitive humidity sensors exhibit remarkable flexibility, high sensitivity (3215.25 pF % RH^−1^), and sustained stability. The sensor's response time, measured from 20% RH to 80% RH, is ≈15.8 s. Furthermore, the researchers evaluated the hysteresis characteristics of the GO‐based humidity sensor by cycling the relative humidity from 10% RH to 90% RH and back to 10% RH, observing low hysteresis during the desorption process, which underscores its efficacy in high humidity conditions. These superior sensing capabilities make LIG/GO‐based sensors suitable for the monitoring of human breath and non‐contact motion. Starting with the integration of the sensor into the mask, the capacitance changes were induced during exhalation and inhalation based on the increase or decrease of moisture in the gas, enabling real‐time monitoring of human respiration. Furthermore, when a moist fingertip was moved from 5 mm to 1 mm from the sensor, the capacitance increased dramatically due to the increase in humidity, highlighting the high sensitivity of the sensor. These findings indicate that the LIG/GO‐based capacitive sensor has promising applications in respiratory monitoring and non‐contact positioning techniques.

To further improve the humidity sensor performance, Fei et al. explored methods to enhance the sensitivity of LIG‐based sensors, focusing specifically on the effects of electrode gap size and GO doping.^[^ ^147^ ^]^ The research utilized picosecond laser ablation on PI film to create a LIG sensor with adjustable electrode spacing. Their findings revealed a direct relationship between the electrode gap size and the sensor's capacitive response, noting that a smaller gap size resulted in a more significant capacitive response. Furthermore, the study observed that at low‐loading GO, the sensor's sensitivity was inversely proportional to the electrode gap size, while at high‐loading GO, the sensitivity showed a direct proportionality to the gap size. This relationship underscores the nuanced impact of GO content on the sensor's performance. Additionally, the researchers identified an optimal GO dosage range, pinpointing that the highest sensitivity, 3862 pF % RH^−1^ (Figure 6b), was achieved when the drop‐coated GO concentration was between 1.45‐1.86 µL m^−2^. These characteristics make it an exceptionally useful tool for applications requiring precise and reliable monitoring of human physiological conditions.

The use of microwave resonant platforms has gained prominence due to their potential for high sensitivity and real‐time detection capabilities.^[^ ^149^ ^]^ These platforms have become particularly advantageous in the development of humidity sensors. Numerous research efforts have been directed toward the improvement of the sensitivity and reduction of the response time of microwave‐based humidity sensors.^[^ ^150^, ^151^, ^152^ ^]^ Despite these advancements, a common challenge identified in the literature concerning microwave humidity sensors is their limited range in humidity detection and issues with reproducibility. These limitations are often attributed to the microwave characteristics of the humidity‐responsive materials used. To address this gap, Adhikari et al. embarked on exploring the microwave humidity sensing properties of LIG.^[^ ^148^ ^]^ LIGs with high porosity 3D network features are patterned onto PI using a CO_2_ laser. This flake was then integrated into the capacitive sensing region of a microwave resonator operating at 1.985 GHz, with the overall layout shown in Figure 6c. The performance assessment of this innovative microwave‐LIG sensor yielded promising results. The sensor could linearly detect a wide range of relative humidity, from 10% to 95% RH. Notably, it exhibited high sensitivity and rapid response and recovery times, clocking in at <6 s. Additionally, the sensor showed minimal hysteresis (0.375% RH) and excellent resolution (0.07% RH). These findings confirm the inherent high‐performance humidity sensing capabilities of LIG in the microwave regime. This research not only contributes to the field of microwave sensing technology but also opens new avenues for the utilization of LIG in diverse sensing scenarios.

In summary, LIG‐based capacitive sensors hold extensive application prospects in human health assessment. Their advantages of flexibility, sensitivity, and biocompatibility enable them to be ideal health monitoring tools that can offer accurate, reliable, and continuous information on physiological parameters, which can significantly help the early diagnosis and treatment of diseases.

### 3.6 Field Effect Transistor

The principle of the field effect transistor (FET), a cornerstone of modern electronics, is based on utilizing an electric field to manipulate the current flow in a semiconductor channel.^[^ ^153^ ^]^ The conductivity between the source and drain is typically regulated by a voltage applied to the gate that produces an electric field. The use of LIG in FET biosensors relies on its excellent electrical conductivity and a porous 3D network structure with increased surface area. This combination is particularly favorable in biomolecular detection.

**Figure 7:**
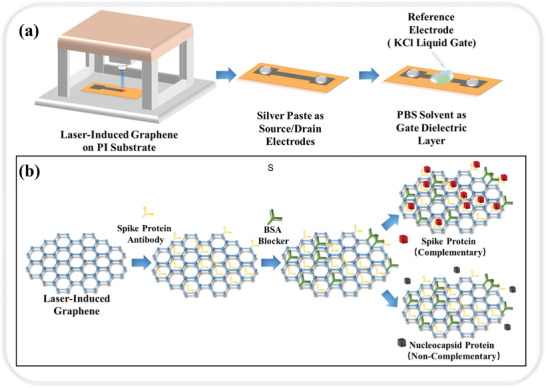
LIG‐based FET sensing. Schematic diagram of a) the preparation process and b) specificity detection of LIG‐based FET sensors. Reproduced under terms of the CC‐BY license.^[^ ^154^ ^]^ Copyright 2021, The Authors, published by MDPI.

LIG‐based FETs have promising applications as biosensors, especially in the detection of biomolecules associated with diseases such as COVID‐19. In this context, LIG‐based FETs can be functionalized with receptors specific to SARS‐CoV‐2, enabling direct and rapid detection of the virus. This approach offers the possibility of not only high sensitivity but also real‐time surveillance, which is crucial in the management of pandemic situations. In the rapidly evolving field of COVID‐19 diagnostics, Cui et al. introduced an innovative biosensing method employing LIG within a field‐effect transistor framework, specifically for the detection of the SARS‐CoV‐2 spike protein, as shown in **Figure** **7**.^[^ ^154^ ^]^ The sensor's design is predicated on the immobilization of specific antibodies on the LIG surface. When these antibodies interact with the SARS‐CoV‐2 spike protein, measurable changes in the electrical properties of the graphene channel are induced, facilitating ultra‐sensitive detection. Remarkably, the sensor demonstrated the capability to detect the spike protein at concentrations as low as 1 pg mL^−1^ in phosphate‐buffered saline and 1 ng mL^−1^ in human serum, showcasing exceptional sensitivity. This study's findings are instrumental in addressing the critical need for rapid, accurate, and low‐cost COVID‐19 testing methods. Unlike conventional RT‐PCR techniques, which are time‐consuming and require sophisticated laboratory infrastructure, this LIG‐based sensor offers a promising alternative for point‐of‐care diagnostics in 15 minutes. Its potential for rapid, on‐site detection of COVID‐19, coupled with the advantages of low‐cost production and ease of use, positions it as a pivotal tool in managing and controlling the pandemic.

While the application of LIG in FET biosensors presents a promising avenue for the sensitive and rapid detection of biomolecules, including those associated with diseases like COVID‐19, it is crucial to acknowledge and address the prevailing challenges faced by LIG‐based FETs. Notably, issues such as stability, preparation cost, and integration performance stand as significant barriers to their widespread adoption. The stability of LIG‐based FETs, which is crucial for reliable long‐term operation, is impacted by environmental factors and operational conditions. Moreover, the cost of preparation, while potentially offset by the scalability of laser‐induced techniques, requires careful consideration of material selection and processing methods to ensure economic viability. Integration performance demands innovative approaches to compatibility and interfacing with existing technologies, particularly in complex electronic systems. An in‐depth understanding and resolution of these challenges are essential for leveraging the full potential of LIG‐based FETs in health monitoring applications.

## 4 Summary and Outlook

In this review, we comprehensively explore the progress, sensing mechanisms, and applications of LIG‐based sensors for health monitoring. Over the past decade, LIG has evolved from a novel laboratory discovery to a versatile sensing material that has shown significant potential for applications, particularly in the field of health monitoring. The distinct sensing mechanisms and versatile applications highlight the potential of LIG in advancing the field of health monitoring. LIG‐based sensors have been able to monitor a wide range of key health indicators through various sensing mechanisms, from biochemical markers to physiological parameters (**Table** **1**). For example, the LIG‐based sensor can detect a variety of biochemical markers, such as uric acid and tyrosine levels, which are critical for assessing kidney function and metabolic status. In addition, LIG has made an important contribution to monitoring hormone levels, such as cortisol levels, to assess stress and mental health. For electrolyte balance monitoring, LIG‐based sensors have demonstrated high sensitivity to changes in the concentration of Na^+^, K^+^, and NH_4_^+^, which are critical to maintaining the healthy functioning of the cardiovascular and nervous systems. LIG technology has also made breakthroughs in pathogen detection, particularly during the COVID‐19 pandemic. LIG‐based sensors can rapidly and accurately detect specific biomarkers associated with COVID‐19 in blood and saliva samples, providing an effective tool for disease diagnosis and surveillance. The application of the LIG‐based sensor is equally compelling in the monitoring of physiological parameters. It can continuously monitor body temperature, providing critical information for early disease diagnosis. At the same time, LIG‐based sensors monitor respiratory conditions such as humidity, frequency, depth, and breathing patterns, which are critical for respiratory health assessment. In addition, they can detect sound and blinking frequency, parameters that have an important role in assessing nervous system function and emotional state. Collectively, the development of LIG technology in the field of health monitoring not only improves the efficiency and accuracy of disease diagnosis and health management but also opens up new possibilities for future healthcare.

**Table 1:** Health monitoring indicators, sensing Mechanisms, and performance of LIG‐based sensors.

Sensing mechanism	Monitored indicator	Health Relevance	Sensitivity	Detection limit	Reference
Electro‐chemistry	Uric acid (sweat)	Kidney function	3.50 µA µm ^−1^ cm^−2^	0.74 µm	[68]
	Tyrosine (sweat)	Metabolic status	0.61 µA µm ^−1^ cm^−2^	3.60 µm	[68]
	Cortisol (sweat)	Stress and mental health	–	0.08 ng mL^−1^	[69]
	C‐reactive protein (serum)	COVID‐19	16.64 nA mL ng^−1^	–	[70]
Potentiometry	Na^+^ (sweat)	Cardiovascular and neurological health	60.2 mV dec^−1^	1 µm	[79]
	Na^+^ (sweat)		63.6 mV dec^−1^	0.1 µm	[80]
	K^+^ (sweat)		59.2 mV dec^−1^	0.01 µm	[80]
	K^+^ (urine)		53.0 mV dec^−1^	0.3 mm	[81]
	NH_4_^+^ (urine)		51.0 mV dec^−1^	0.1 mm	[81]
Thermo‐electricity	Temperature	Critical information for early disease diagnosis	−2.8 × 10^−3^ °C^−1^	–	[88]
			−1.5 × 10^−3^ °C^−1^	–	[92]
			1.4 × 10^−3^ °C^−1^	–	[93]
			−4.5 × 10^−4^ °C^−1^	0.2 °C	[94]
Piezo‐resistivity	Airflow	Breathing patterns	0.11 s m^−1^	0.0023 m s^−1^	[129]
	Strain	Blinking movements	1242	0.025%	[137]
	Pressure	Electronic skin	−34.15 kPa^−1^	0.036 kPa	[141]
Capacitance	Humidity	Respiratory conditions	3215.25 pF % RH^−1^	–	[146]
		Skin moisture	3862 pF % RH^−1^	–	[147]
Field effect transistor	Spike protein (serum)	COVID‐19	–	1 ng mL^−1^	[154]

The focus of future research can be centered on the following aspects. First, further improvement of sensor sensitivity. Although LIG‐based sensors have demonstrated high sensitivity in many applications, specific application areas (such as airflow sensing) still require further sensitivity enhancement. Future research could focus on effectively resolving the structure‐performance relationship to enhance the sensitivity of LIG sensors through precise tuning of the microstructure, selection of admixtures, and incorporation of innovative technologies for more accurate monitoring. Second, reduction of interference between multi‐physics fields. In health monitoring, multi‐physical fields (like temperature, humidity, and pressure) tend to affect each other, posing challenges to the accuracy and reliability of sensors. Future research needs to address how to effectively reduce the interactions between these physical fields to achieve interference‐free multi‐parameter high‐precision monitoring. This may involve complex signal processing algorithms and more advanced sensor designs. Third, the integration of LIG‐based sensors. Currently, LIG‐based sensors suffer from insufficient integration to achieve all‐round health monitoring. The future goal is to achieve a more advanced level of integration through rational modulation of physical (like 3D structural design) and chemical (like optimization of substrate materials or admixtures) properties. This includes self‐powering, multi‐parameter monitoring, and wireless data transmission. Ideal LIG‐based sensors will be able to monitor multiple health indicators simultaneously through diverse sensing mechanisms, along with self‐powering and wireless communication capabilities.

In summary, the future development of LIG technology in health monitoring will focus on improving sensor sensitivity, reducing interference between multiple physical fields, and achieving a higher degree of integration. This will involve innovations in the areas of materials science, sensor engineering, and data analytics. In addition, LIGs can be combined with other biosensors and microelectronic devices to form integrated multifunctional sensors for monitoring temperature, blood pressure, oxygen saturation, and other physiological parameters in the human body.^[^ ^77^, ^155^, ^156^ ^]^ The ability of LIG to adapt to various sensing mechanisms and its integration into multifunctional devices pave the way for innovative solutions in personalized health monitoring and environmental diagnostics. As these challenges are gradually overcome,^[^ ^157^ ^]^ the LIG‐based sensors are expected to play a key role in real‐time, all‐encompassing health monitoring systems, providing more accurate and reliable technical support for personal health management and clinical diagnosis.

## Conflict of Interest

The authors declare no conflict of interest.
